# Intravenous contrast medium extravasation: systematic review and updated ESUR Contrast Media Safety Committee Guidelines

**DOI:** 10.1007/s00330-021-08433-4

**Published:** 2022-02-17

**Authors:** Giles Roditi, Nadir Khan, Aart J. van der Molen, Marie-France Bellin, Michele Bertolotto, Torkel Brismar, Jean-Michel Correas, Ilona A. Dekkers, Remy W. F. Geenen, Gertraud Heinz-Peer, Andreas H. Mahnken, Carlo C. Quattrocchi, Alexander Radbruch, Peter Reimer, Laura Romanini, Fulvio Stacul, Henrik S. Thomsen, Olivier Clément

**Affiliations:** 1 Department of Radiology, Glasgow Royal Infirmary, Glasgow, UK; 2 Department of Radiology, Leiden University Medical Center, Leiden, The Netherlands; 3 University Paris-Saclay, AP-HP, University Hospital Bicêtre, Service de Radiologie, BioMaps, Le Kremlin-Bicêtre, France; 4 Department of Radiology, University Hospital Trieste, Trieste, Italy; 5 Department of Clinical Science, Intervention and Technology, Unit of Radiology, Karolinska Institutet and Department of Radiology, Karolinska University Hospital in Huddinge, Stockholm, Sweden; 6 Université de Paris, AP-HP, Groupe Hospitalier Necker, DMU Imagina, Service de Radiologie, Paris, France; 7 Department of Radiology, Northwest Clinics, Alkmaar, The Netherlands; 8 Department of Radiology, Landesklinikum St. Pölten, St. Pölten, Austria; 9 Department of Diagnostic and Interventional Radiology, Marburg University Hospital, Marburg, Germany; 10 Imaging Center, Unit of Diagnostic Imaging and Interventional Radiology, Università Campus Bio-Medico Di Roma, Rome, Italy; 11 Department of Radiology, Clinic for Diagnostic and Interventional Neuroradiology, Bonn, Germany; 12 Department of Radiology, Institute for Diagnostic and Interventional Radiology, Klinikum Karlsruhe, Karlsruhe, Germany; 13 Department of Radiology, ASST Cremona, Cremona, Italy; 14 Department of Radiology, Ospedale Maggiore, Trieste, Italy; 15 Department of Radiology, Copenhagen University Hospital Herlev, Copenhagen, Denmark; 16 Université de Paris, AP-HP, Hôpital Européen Georges Pompidou, DMU Imagina, Service de Radiologie, 20 Rue LeBlanc, 75015 Paris, France

**Keywords:** Contrast media, Extravasation of diagnostic and therapeutic materials, Risk factors, Prevention and treatments

## Abstract

**Need for a review:**

Guidelines for management and prevention of contrast media extravasation have not been updated recently. In view of emerging research and changing working practices, this review aims to inform update on the current guidelines.

**Areas covered:**

In this paper, we review the literature pertaining to the pathophysiology, diagnosis, risk factors and treatments of contrast media extravasation. A suggested protocol and guidelines are recommended based upon the available literature.

**Key Points:**

*• Risk of extravasation is dependent on scanning technique and patient risk factors.*

*• Diagnosis is mostly clinical, and outcomes are mostly favourable.*

*• Referral to surgery should be based on clinical severity rather than extravasated volume.*

**Supplementary Information:**

The online version contains supplementary material available at 10.1007/s00330-021-08433-4.

## Introduction

Contrast media extravasation (CMEX) is a complication where there is leakage of intravenously administered contrast agents (either iodine or gadolinium-based), into the surrounding soft-tissues [1]. This can vary in severity from minor discomfort to compartment syndrome, skin ulceration and necrosis. A recent observational study of 142,651 participants undergoing CT scans showed a CMEX incidence of 0.23% [2] whilst a systematic review by Behzadi et al. accounting for 17 studies in 1,104,872 patients found CMEX rates of 0.2% [3]. Rates of serious CMEX seem much lower, only described in case reports and case series [4–7] although this probably reflects under-reporting.

CMEXs are thought to be one of the most frequent adverse events in radiology but are much less studied than others such as contrast-associated acute kidney injury [8–10]. Whilst CMEX does not usually lead to significant morbidity, the rare, serious complications of compartment syndrome, skin ulceration and tissue necrosis are important to recognise [4–6]. Furthermore, even what may be clinically regarded as minor CMEX will be perceived as important by the patient and contribute to feelings of dissatisfaction at a stressful time since “something has gone wrong” with their care.

Previous guidelines around CMEX from the Contrast Media Safety Committee (CMSC) of the European Society of Urogenital Radiology (ESUR) published in 2002 related to older contrast injection protocols [1]. Since then, several prospective studies investigating CMEX risk factors and management as well as systematic reviews have been published [2, 3, 11–13]. In this paper, we aim to inform update of the CMSC guidelines by performing a systematic review and provide recommendations.

## Methodology

**Fig. 1:**
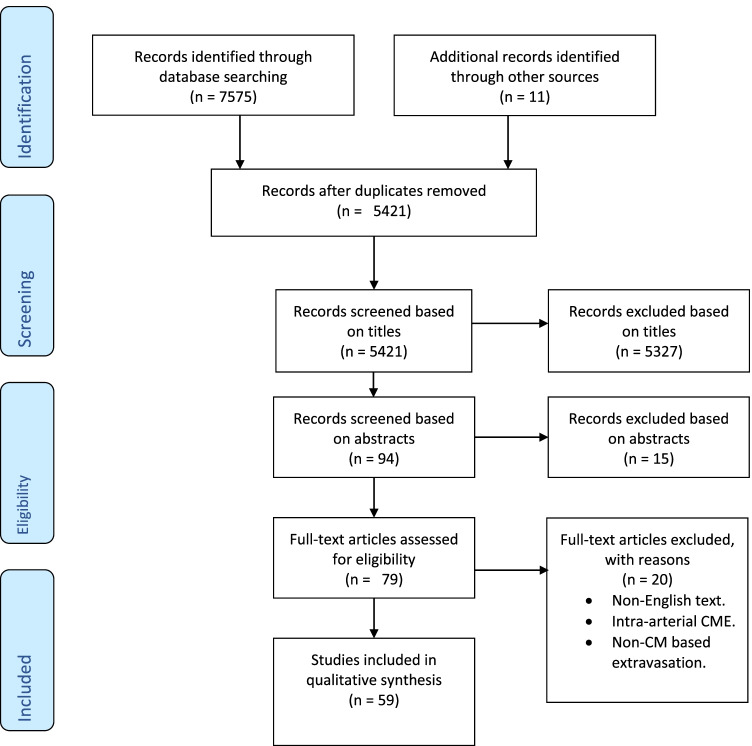
Search and selection procedures

Authors prepared seven clinical questions in Patient–Intervention–Comparison–Outcome (PICO) format [14]. A systematic literature search of PubMed and Scopus from 1 January 1990 through to 6 February 2021 with MeSH terms as described in the search strategy (Appendix 1) was performed by a radiology trainee (N.K.) with previous meta-analysis experience and a radiology consultant (G.R.). Abstracts were reviewed for relevance, references scrutinised and cross-referenced. Only articles in English pertaining to intravenous CMEX in context of CT and MRI were considered. References from previous CMSC guidelines were included. CMEXs associated with contrast-enhanced ultrasound were excluded as no cases have been reported in the literature [15–17]. Studies assessing intra-arterial CMEX and non-contrast media-based extravasation were also excluded (Fig. 1 flow-chart of article selection and final number of articles).

Risk of bias of each study included was graded according to National Institutes of Health (NIH) study quality assessment tools. The strength of recommendation of different risk factors, diagnosis, detection and management of CMEX were graded according to OCEBM Levels of Evidence Working Group “The Oxford 2011 Level of Evidence” (Appendices 2 and 3). Characteristics, risk of bias and levels of evidence for each included study are outlined in Appendix 4.

Studies were synthesised and summarised narratively.

### What are the pathophysiologic mechanisms of CMEX?

Three main processes underpin the mechanisms of CMEX [18]. The first model describes fluid escaping into perivascular tissue through extraluminal dislocation or break of the catheter tip. The second model describes a leak through the puncture site of a correctly placed cannula leading to extravasation. The third model envisages shear stresses and total pressure from the jet at the vessel wall directly leading to vessel disruption and extravasation [19].

The degree to which tissue damage occurs due to CMEX is dependent on the CM used (osmolality, cytotoxicity and extravasated volume) plus location of the cannula; more damage occurs with involvement of tight sub-fascial compartments compared to looser subcutaneous layers [20]. An acute inflammatory response which peaks up to 48 h after injection can be followed by several weeks of chronic inflammation [21]. However, the majority resolve in 2–4 days with resorption of CM extravasate thought to be primarily by the lymphatic system [13].

Consequently, much research has been based on preventing cannula dislodgement and exploring the impact of variables that increase risk of leakage from the puncture site.

### How to detect extravasation?

With CMEX contrast escapes and infiltrates the interstitium during injection. This is mostly a clinical diagnosis and routine use of imaging is not indicated for its detection.

Non-physicians are usually first responders to CMEX, i.e. radiology technicians/radiographers and healthcare support workers. Hence, it is recommended that imaging departments follow a protocol which allows identification of at-risk patients, easy detection of CMEX, awareness of monitoring needs and effective management of extravasation by a wide range of staff [22]. As an excerpt from multiple publications, the following algorithm is proposed (Table 1). Outpatients with extravasation should be kept under observation in the department until a physician is satisfied that signs and symptoms are resolving such that intervention will not be needed and the patient allowed home.

**Table 1:** Recognition and diagnosis of contrast media extravasation

		Grade of recommendation*
Instruct the patient to report any pain or swelling, during or after injection		D
Observe for signs of extravasation directly both during and following injection plus, and directly palpate the cannula insertion site		C
During Contrast media infusion observe for any alerts on the contrast injection system and observe the patient for signs of distress. Observe monitoring scans for expected contrast arrival and completed scans for enhancement		B
Mark out affected area when contrast media extravasation occurs		B
Assess severity of any extravasation:		
Mild	Minor erythema or swelling, no skin changes	
Moderate	Skin blistering, progressive oedema and/or ulceration. These will require close monitoring and physician assessment is advised to assess for any neurovascular compromise by checking peripheral pulse and sensation distal in the affected limb	
Severe	Any neurovascular compromise, signs of tissue necrosis or compartment syndrome. This would require urgent surgical attention e.g. emergency fasciotomy	

^*^Grade of recommendation (see Appendix 2)

We summarise three main ways of detecting CMEX in Table 2.

**Table 2:** Detection of contrast media extravasation

1. *Direct Patient Observation:* Technician/radiographer in room or video monitoring as well as self-reporting by the patient. Observation of monitoring scans for expected contrast arrival and completed scans for enhancement. 2. *Pressure Monitoring:* Power-injectors for contrast media (CM) administration now have pressure monitoring systems with graphic displays and flow profile previews which can automatically or semi-automatically stop the CM injection if increased resistance or faults within the system are encountered. One of the promoted benefits of this technology is CM extravasation prevention/minimisation although no published studies have specifically explored their impact to date. 3. *Extravasation detection accessories:* Some vendors have developed sensors to detect CM extravasation amongst other capabilities.	

Many vendors have developed Extravasation Detection Accessories (EDA) which are sensors that allow detection and interruption of automated injection in the event of an extravasation. Dykes et al. demonstrated the use of EDA resulted in smaller volumes of CMEX when they occurred compared to when no EDA was utilised (1,085 CMEX events in 454,497 CT exams across 58 radiology practices) [23]. However, incidence of CMEX did not change and importantly some large volume extravasations still occurred with 28 patients suffering 50–99 ml of CMEX and 4 patients experiencing > 100 ml of extravasate (unlike studies by Powell et al. and Birnbaum et al.). However, with multiple centres, it was difficult to compare the performance of different EDAs against each other since the centres used different EDAs (types not specified) [23].

One quality improvement project and three case-series have investigated EDA use; each assessed a different device [24–27]. Two studies only published preliminary results and did not specify sensitivity or specificity [28, 29]. These devices rather than fully preventing extravasation are designed to detect it early and so prevent a mild extravasation from becoming moderate or even severe. A summary of the clinically tested EDA devices is provided in Appendix 5.

### Is documentation of extravasation needed?

Proper documentation of CMEX is crucial [12]; at the patient level, this allows early recognition of deterioration and aids interpretation across a broad range of healthcare staff. More broadly, documentation at an institutional level allows auditing which can improve local working practices. Dykes et al. showed that the rates of observed CMEX reduced as a result of multi-institution audit; however, they did not impact the volume of extravasate when extravasation did occur [23].

With severe extravasations, imaging documentation may be helpful [30]. Plain radiographs with 2 orthogonal views, 2-plane CT topogram or indeed cross-sectional images with the CT or MRI scanner before removing the patient from scanner table are recommended to assess for compartmentalisation (subfascial vs subcutaneous) and extent of extravasate [21, 31].

### What are the risk factors for extravasation?

#### Technique related

##### Peripheral IV cannula type, size and location

Six studies evaluated the influence of the physical properties of cannulae; none were prospective randomised trials. A pseudo-randomised trial compared between a fenestrated 20G and a non-fenestrated 18G cannula which showed no effect on CMEX rates or volume [32]. Furthermore, retrospective cohort studies have shown that extravasation rates were higher with 22G compared to 20G and 18G cannulas, but no significant difference between 18 and 20G cannulas [33–35]. In addition, these studies have reported no difference in extravasate volume between the use of different cannula sizes. In a paediatric prospective cohort study, there was no effect of cannula size on CMEX [36]. Location of the peripheral cannula was an important risk factor in three studies with higher rates of extravasation when placed in a vein in the dorsum of hand compared to an antecubital fossa vein. However, higher volume of extravasate was observed with injections in antecubital fossa versus hand veins despite the higher incidence at the hand/wrist, likely related to use of higher flow rates and delayed recognition of CMEX at the antecubital fossa. Overall, injection sites in the lower limbs and small distal veins are less optimal.

##### CVCs, PICCs and power injectable ports

Central venous catheters (CVC—tunnelled or non-tunnelled), totally implantable vascular access devices, haemodialysis catheters and peripherally inserted central catheters (PICCs) are increasingly used for patients in critical care, on chemotherapy or long-term antibiotics. Of course these patients often require regular cross-sectional imaging with IV contrast. Power injector compatible versions of these have been shown to be safe [37], with a low 1% reported risk of adverse incident. Each type of catheter has maximal flow rate and pressure limits detailed by the manufacturer. However, there remains variability in practice and persistent concern regarding complications due to potentially high pressures achieved using pump injections of CM and how very few catheters have been studied for use with power injectors [38, 39]. Although rare, extravasation from central catheters can lead to significant morbidity, i.e. mediastinal extravasation, haematoma and cardiac arrhythmias.

Prior to the advent of power-injectable CVCs, the Medical and Healthcare Products Regulatory Agency (MHRA) in the UK published recommendations regarding rates and volumes for CM injections. However, these have been withdrawn and current advice is to follow specific manufacturer guidance. Plumb and Murphy recommended a maximum flow rate of 2.0 ml/s for CVC and to use the distal lumen if a multi-lumen CVC is in place [37]. A CT topogram is a good method to quickly evaluate catheter tip positioning post CM administration. Overall, extravasation injury is extremely rare with the main concern pertaining to mispositioning and tube integrity. Power injectable TIVADs have a growing role; a retrospective study did not find any incidence of CMEX in 307 patients studied over 4 years with injection rates of 3–5 ml/s employed. Although the evidence base for the use of these power injectable ports is limited, there has been no CMEX demonstrated [40]. Another retrospective study found similar findings [41] whilst Rigsby et al. also found pressure-limited power injection through central lines in children to be safe (no complications, 63 patients aged 0.3–22 years) [42].

##### Power injection compared to manual injection via peripherally inserted IV cannula

Sinan et al. did not find any significant difference in extravasation rates in patients receiving CM via power injection vs. manual injection [34]. A study by Barrera et al. of power injectors in children also found low rates of extravasation and long-term injury [43]. Preparation of the injector system is key to minimise the risk and departments should follow a standard protocol, i.e. correct alignment and clearing of syringe and pressure tubing.

##### Infusion rate and volume of CM

Six studies have assessed rate and volume impact on CMEX. A randomised controlled trial by Kok et al. demonstrated no significant difference in CMEX when assessing different flow rates (8.3 ml/s, 6.7 ml/s and 5.4 ml/s) [44]. However, different CM were used to ensure equivalent iodine delivery rate and load remained constant. Furthermore, a major confounding variable was that catheter size was not kept constant. Findings from other research groups [34, 34, 45, 46] are similar with the caveat that all have confounding factors of different cannula sizes and type of CM used. Whilst Wienbeck et al. found a statistically significant increase of extravasate volume with the use of high flow rates, Moreno et al. showed the converse of reduced extravasate volumes with higher flow rates [46]. The use of EDAs in this study appeared to reduce volumes of extravasate at higher rate injections.

##### Cannula insertion technical factors

A non-randomised retrospective study found higher rates of extravasation in patients who had ultrasound-guided cannula insertion (3.6%) vs. standard insertion (0.3%). However, the numbers of patients in each group were drastically different—364 in the ultrasound-guided cannula inserted group and 896 in the standard cannula inserted group (2% of a random sample of 40,143 patients). The difference in results in this study is likely due to confounding variables; i.e. prior failure of standard insertion and with deeper veins there is potentially a shorter length of intravascular cannula hence greater potential for dislodgement upon injection [47]. The type of healthcare worker who inserts the IV cannula was investigated by Sinan et al. which showed that CMEX rates were higher when inserted by non-radiology staff (0.3%) as compared to radiology staff (0.2%). However, this difference did not demonstrate statistical significance and a number of confounders could explain this difference [34]. Another study evaluated extravasation between newly inserted cannula vs. use of an existing cannula for CM injection with a difference in rates and a reduced volume of extravasate observed with those freshly sited (40.6 ml vs. 63.1 ml, *p* = 0.0005) [46], despite older cannulae being checked for flush efficacy. Therefore, consideration should be given to placing a new cannula for at-risk patients to reduce potential impact of any extravasation.

##### Chemical properties of CM

Three main contrast media properties have been studied in relation to CMEX: osmolarity, charge and viscosity.

Osmolarity—A relationship has been demonstrated between higher osmolarity CM (600, 1500 and 2100 mOsm/kg H_2_O) and cellular lysis which is thought to be a factor in the degree of tissue damage caused by extravasation. This has only been studied in animal models where old ionic CM (such as sodium and meglumine ioxithalamate) was used [48, 49]. Human clinical studies with non-ionic CM are lacking.

Ionicity—Charge can influence extravasation in that ionic CMs are thought to increase complications; however, ionic IBCMs are no longer used for intravenous studies. Studies have shown that non-ionic IBCMs (such as Iopamidol 300 and 370, and iohexol 300 and 350) are well tolerated in humans [30, 49, 50]. On the other hand, most GBCAs are ionic so this can potentially play a role in the severity of extravasation injury although volumes used are much lower and again human clinical studies are lacking.

Viscosity—This is thought to influence the likelihood of extravasation occurring. A computational fluid dynamics study by Sakellariou et al. demonstrated the potential for increased incidence of extravasation with more viscous CM, especially in CT angiography when performed with smaller peripheral cannulas [19]. A viscosity of > 9.4 mPa·s demonstrated increased risk of extravasation in the study by Hwang et al. [2]. An inverse relationship exists between viscosity and temperature in liquids; hence, warmed CMs are less viscous and offer lower resistance. Davenport et al. evaluated the role of warming CM to 37 degrees vs. CM at ambient temperature in > 20,000 patient contrast injection trialled with two contrast media. There was no significant difference in CMEX incidence between warmed versus ambient temperature Iopamidol 300. However, the higher viscosity Iopamidol 370 had an increased incidence of CMEX that was reduced (becoming similar to that with the lower viscosity CM) when the CM was warmed. There was, however, no effect on the volume of extravasate when CMEX occurred [51]. A recent study evaluating safety of Iomeprol 400, when warmed to 37 degrees, reported CME rate of 0.71% out of a total of 3,514 injections, with site and/or size of cannula not influencing CMEX rates [52]. However, the authors found inability to aspirate blood through the cannula significantly correlated with incidence of CMEX.

Overall, this suggests that using CM with lower viscosity and/or warming CM and/or diluting/mixing CM with saline has a preventative role in CMEX.

##### Gadolinium based contrast agents

There is much less data on rates of CMEX for MRI; the incidence of CMEX is reported as approximately 0.06% with no serious complications described—likely due to low infusion rates and lower CM volumes compared to IBCM uses. Theoretically, the extravasation of GBCAs could lead to oedema, necrosis or haemorrhage, potentially exacerbated due to their ionicity and higher osmolarity when compared to IBCM, although this does not seem to be borne out in practice [3, 48].

#### Patient-related factors

Based on a meta-analysis of 356,582 patients by Ding et al., females and older patients (> 60 years) are at greater risk of developing CMEX; however, gender and age have no impact on the volume of CM extravasation when it does occur [11]. In-patients are at an increased risk compared to those having outpatient scans, especially intravenous drug users and patients with recent hospitalisation [11]. Further risk factors include those patients with an inability to communicate, fragile veins, compromised venous and/or lymphatic drainage and obesity [1].

It is suggested based on the risk factors discussed in Table 3 that the measures outlined in Table 4 will greatly reduce the chances of extravasation occurring and minimise severity when it occurs.

**Table 3:** Risk factors

Technique	Patient
• Less optimal injection sites including lower limb and small distal veins • Large volume of contrast medium • High osmolarity contrast media • Viscous contrast media	• Inability of patient to communicate • Fragile or damaged veins • Compromised lymphatic and/or venous drainage • Obesity

**Table 4:** Preventative and minimisation measures

	Grade of recommendation*
Meticulous cannula insertion technique using an appropriate size upper arm vein is preferred	C
An appropriately sized cannula for the vein and anticipated flow rate	B
Test injection with saline prior to contrast administration	D
Warming of the contrast medium, especially for higher viscosity compounds	B
Minimising the volume of contrast administered based upon the indication and patient size	B
Use of correct flow rates and pressures appropriate to the specific catheter, especially when using central venous catheters	B
Effective detection protocol which allows early diagnosis, this ranges from direct observation to considering use of extravasation detection accessories in high-risk patients	B

^*^Grade of recommendation (see Appendix 2)

### What are the proposed treatments?

The untreated sequelae of severe CMEX are as follows: increased intra-compartmental pressure (compartment syndrome), with subsequent risk of ischemia due to venous congestion and low arterial gradient causing disproportionate necrosis, severe neurovascular compromise or even limb loss. Treatments to avoid these serious complications can be divided into passive conservative measures and more active therapies (Table 5).

**Table 5:** Treatment of contrast media extravasation (CMEX)

	Mechanisms and discussion of evidence
***Conservative Treatments***	
Aspiration of contrast whilst cannula still in place prior to removal.	This reduces the volume of contrast extravasate and reduce pressure [57].
Raise the affected limb if possible.	Minimise oedema by reducing hydrostatic pressure and promoting drainage [58].
Cooling of the region.A cold compress 15 to 60 minutes three times per day for a period of 3 to 4 days [1].	Anti-inflammatory effect via vasoconstriction and is widely recommended when treating CMEX [1, 13, 59].
Warming of the region.	Controversially, some think that cooling can delay resorption of extravasate, and warming can lead to vasodilation hence increasing contrast media (CM) resorption. Hastings-Tolsma et al. conducted studies with saline, assessing extravasation by the effect of both warming and cooling extremities [60]. No significant difference was observed between groups in terms of surface area induration, or evidence of extravasate taking longer to resorb when warm solution was applied.
Heparin ointment dressing with cooling (where the dermis is intact).	Anecdotal use has been suggested in a recent review paper by Mandlik et al. [12].
Topical non-steroidal anti-inflammatory drugs (NSAIDs).	Evidence only pertains to the analgesic effects on acute pain and not specifically extravasation [61].
***Invasive treatments***	
Hyaluronic acid injection (HYLA). Dose of between 5–250 Units is thought to be most effective [15].	This mucopolysaccharide is injected directly into the site of CMEX and is thought to work by enzymatically cleaving structures of the interstitium thus promoting resorption into vessels and lymphatics. Limited evidence supporting its use [62]. Indeed, some data does not support its use as some animal models have shown an increased inflammatory response [63]. Overall, not considered routine treatment (only off-label use e.g. inoperable patients with compartment syndrome due to CMEX).
Aspiration & irrigation: essentially “wash-out” using stab incisions around the area of concern under local anaesthetic and extravasate aspirated with blunt suction cannulas. This is followed by irrigation (performed within 6 hours).	There is variation as to the exact technique, based on a retrospective study by Gault in 96 patients with extravasation, 44 were successfully treated [64]. However, only 1 patient had a CMEX (others being chemotherapy agents etc.) meaning it may be less applicable to CMEX. Further case series of 11 patients by Vandeweyer et al. described successful use, although this was with high osmolarity, ionic agents [65]. Overall, this is a mechanistically plausible method but without strong evidence base for routine use.
Manual squeezing technique: manual expression of extravasate after various punctures/stab incisions (e.g. 5–10 stabs with 18G needle).	Study by Tsai et al. of 8 cases who developed vascular compromise with 50 - 80 ml of non-ionic, low osmolarity extravasate demonstrated satisfactory healing using this method [66]. A similar study by Kim et al. with 23 cases (no control group) of extravasate > 50 ml also showed satisfactory response after 1 week follow-up although there was immediate temporary mild blistering [67]. A similar technique whereby multiple stab incisions were made for large volume extravasations was found to be successful in a case report by Raveendran et al. [68] Similar to other techniques, limited data available to support use of this, but the simplicity is attractive and more comparative data would help assess efficacy.
Fasciotomy and compartment release	Considered the definitive surgical treatment when a CMEX is complicated by neurovascular compromise or compartment syndrome. A retrospective study by Fallscheer et al., identified seven patients required fasciotomy [54]. Delay to refer to plastic surgery by >300 minutes is the greatest risk factor contributing to complications post-operatively.

Studies assessing conservative methodologies to treat extravasation events are few and not of robust quality in terms of applicability for radiology, i.e. laboratory-based studies or investigations of extravasation of cytotoxic drugs rather than contrast media. Most recommendations are based on “good clinical practice”. The aim of conservative measures is to reduce the morbidity associated with CMEX. The evidence base for use of invasive treatments is limited with mostly retrospective studies, small case numbers, lack of control groups, data based on older CM or even not CMEX.

It is important that clear instructions to the patient who has suffered CMEX are given regarding when to seek additional medical care if there are worsening symptoms. A patient information leaflet is recommended, available in the languages appropriate for the institution. Patients should be warned about red flag signs and symptoms which are as follows:

- Increased swelling or pain
- Increased redness
- Change in sensation in the affected limb
- Skin ulceration or blistering

In Table 6, we outline a suggested protocol for the management of contrast media extravasations.

**Table 6:** Suggested protocol for management of contrast media extravasation

Grade of recommendation*	
Conservative	
• Stop injection and scan—classify as mild, moderate, or severe	D
• Accurate documentation, demarcate area affected and consult responsible physician	C
• Mild cases: limb elevation, ice packs, monitor patient 2–4 hourly. If improving, then discharge. If no improvement, then requires surgical opinion	C
• Radiographic documentation for moderate and severe cases—two orthogonal views or cross-sectional imaging can help assess compartmentalization and extent of extravasation	C
• Record extravasation as a complication in radiology report and the local incident reporting system	C
• Patient information leaflet should be given to patient	C
• Follow-up appointment, if necessary	D
Active	
• If severe injury (e.g. neurovascular compromise, compartment syndrome, tissue necrosis) suspected then urgently seek advice of a surgeon	B
• Surgical opinion also recommended for extravasate > 150 ml	C

^*^Grade of recommendation (see Appendix 2)

### When to get a surgical consult?

Compartment syndrome is the most serious among the consequences of CMEX. It is extremely rare with less than 12 cases reported in a recent literature review however must be suspected if patient complains of severe pain and/or neurovascular compromise [53]. Signs which would necessitate fasciotomy being painful active flexion, passive extension, neurosensory disturbance and increasing swelling. Locations which are most at risk are volar forearm and the hand. Whilst a radiologist can attend to mild cases, in more severe cases, a surgeon should be urgently consulted, and fasciotomy should be performed within 2 h to avoid muscle necrosis and nerve damage. Surgery performed within 90 to 300 min has been reported to conserve all extremities with favourable prognosis [54]. Sbitany et al. in a study of 102 cases of CMEX used a threshold of 30 ml extravasate as a referral prompt [55] and none required surgery, even in the 10% with > 100 ml CMEX volume. Wang et al. reported similar findings where even extravasate volumes of up to 150 ml of non-ionic IBCM were conservatively managed, suggesting no discrete threshold extravasate volume should be set [56]. Dykes et al. suggest that larger extravasated volumes correlate with moderate-to-severe injuries, but the data does not support using a volume threshold to determine surgical referral or more aggressive management [23]. Nevertheless, in unusual cases with very large volumes of extravasate (> 150 ml), the consensus remains that surgical consult is appropriate.

Overall, the decision to refer for surgical intervention should be a clinical one—i.e. based on red flag signs and symptoms, rather than arbitrary CMEX volumes.

## Conclusion

This review highlights the key up-to-date evidence pertaining to CMEX summarising the important risk factors and a systematic approach to management. Whilst this review has been all encompassing, there are some limitations. Heterogeneity of the studies included in the paper have made performing a meta-analysis tricky and often difficult to compare data across different types of studies. Most of the studies are retrospective and with rates of CMEX being generally low, there are inadequately statically powered studies. There have been technological changes in CT and MRI, especially with use of non-ionic CM, increased understanding of the risk factors and use of EDAs as well as systems which halt injection if problems are encountered during infusion, all of which inform update on the previous guidelines. However, there remain important areas where further research would be merited. There is no data available on patient experience of CMEX and this is an important impact to explore as the patient may refuse to have CM in the future. Long-term follow-up of patients after CMEX and prospective trials with CMEX interventions are also not well-researched. Important questions to ask are appropriate time interval when to re-scan after a CMEX, impact on workflow and cost implications.

## Supplementary Information

Below is the link to the electronic supplementary material.

Supplementary file1 (DOCX 27.1 KB)

Supplementary file2 (DOCX 27.3 KB)

Supplementary file3 (DOCX 27.5 KB)

Supplementary file4 (DOCX 90.8 KB)

Supplementary file5 (DOCX 33.3 KB)

## Abbreviations

CM: Contrast media
CMEX: Contrast media extravasation
CT: Computed tomography
EDA: Extravasation detection accessory
GBCA: Gadolinium-based contrast agent
IBCM: Iodine-based contrast media
IV: Intravenous
MeSH: Medical subject headings
MRI: Magnetic resonance imaging
OCEBM: Oxford Centre for Evidence-Based Medicine*

## Declarations

### Guarantor

The scientific guarantor of this publication is Olivier Clément.

### Statistics and biometry

One of the authors has significant statistical expertise (Ilona Dekkers).

### Methodology

• retrospective
