# Supplementary material for: Intravenous contrast medium extravasation: systematic review and updated ESUR Contrast Media Safety Committee Guidelines
Source: Eur Radiol. 2022 Feb 17;32(5):3056–66. doi: 10.1007/s00330-021-08433-4 (PMC9038843; doi:10.1007/s00330-021-08433-4)
Supplement: Supplementary file 1 — Supplementary file1 (DOCX 27.1 KB) [file 330_2021_8433_MOESM1_ESM.docx]

**Appendix 1: Search strategy**

| **Database: PubMed** |
| --- |
| DATE: no limits/last searched 06th February 2021 |
| Total: 5345 |
| Strategy: (““contrast media” AND “extravasation” OR “contrast media extravasation” OR “paravasation” was performed. Further search terms combined with above search included “central catheter”, “central line”, “central venous”, “PICC” and “port-a-cath”. |
| **Database: Scopus** |
| DATE: last searched 06th February 2021 |
| Total: 2230 |
| Strategy: (“TITLE-ABS-KEY ( "Contrast media" AND "extravasation" ) AND ( LIMIT-TO ( PUBSTAGE , "final" ) ) AND ( LIMIT- TO ( DOCTYPE , "ar" ) OR LIMIT-TO ( DOCTYPE , "re" ) ) AND ( LIMIT-TO ( EXACTKEYWORD , "Contrast Medium Extravasation" ) ) AND ( LIMIT- TO ( LANGUAGE , "English" ) ) AND ( LIMIT-TO ( SRCTYPE , "j" ) ) |
