# Supplementary material for: Intravenous contrast medium extravasation: systematic review and updated ESUR Contrast Media Safety Committee Guidelines
Source: Eur Radiol. 2022 Feb 17;32(5):3056–66. doi: 10.1007/s00330-021-08433-4 (PMC9038843; doi:10.1007/s00330-021-08433-4)
Supplement: Supplementary file 2 — Supplementary file2 (DOCX 27.3 KB) [file 330_2021_8433_MOESM2_ESM.docx]

**Appendix 2: Grades of Recommendation. Adapted from OCEBM Levels of Evidence Working Group*. “The Oxford Levels of Evidence 2”.[1]**

| **Grade** | **Description** |
| --- | --- |
| A | Consistent level 1 studies |
| B | Consistent level 2 or 3 studies or extrapolations from level 1 studies |
| C | Level 4 studies or extrapolations from level 2 or 3 studies |
| D | Level 5 evidence or troubling inconsistent or inconclusive studies of any level |
| ** OCEBM Levels of Evidence Working Group = Jeremy Howick, Iain Chalmers (James Lind Library), Paul Glasziou, Trish Greenhalgh, Carl Heneghan, Alessandro Liberati, Ivan Moschetti, Bob Phillips, Hazel Thornton, Olive Goddard and Mary Hodgkinson* | |

1. OCEBM Levels of Evidence. (2016) In: CEBM. https://www.cebm.net/2016/05/ocebm-levels-of-evidence/. Accessed 10 Nov 2019
