# Supplementary material for: Intravenous contrast medium extravasation: systematic review and updated ESUR Contrast Media Safety Committee Guidelines
Source: Eur Radiol. 2022 Feb 17;32(5):3056–66. doi: 10.1007/s00330-021-08433-4 (PMC9038843; doi:10.1007/s00330-021-08433-4)
Supplement: Supplementary file 3 — Supplementary file3 (DOCX 27.5 KB) [file 330_2021_8433_MOESM3_ESM.docx]

**Appendix 3: Levels of Evidence. OCEBM Levels of Evidence Working Group*. “The Oxford Levels of Evidence 2”.[1]**

| **Levels** | **Description** |
| --- | --- |
| 1a | Systematic reviews/meta-analysis of randomised controlled trials |
| 1b | Individual randomised controlled trials |
| 2a | Systematic reviews/meta-analysis of cohort studies |
| 2b | Individual cohort study or low quality randomised controlled trial |
| 3a | Systematic review/meta-analysis of case-control studies |
| 3b | Individual case-control study |
| 4 | Case series/case-reports (and poor-quality cohort and case-control studies) |
| 5 | Expert opinion without explicit critical appraisal, or based on physiology, bench research or “first principles” |
| ** OCEBM Levels of Evidence Working Group = Jeremy Howick, Iain Chalmers (James Lind Library), Paul Glasziou, Trish Greenhalgh, Carl Heneghan, Alessandro Liberati, Ivan Moschetti, Bob Phillips, Hazel Thornton, Olive Goddard and Mary Hodgkinson* | |

1. OCEBM Levels of Evidence. (2016) In: CEBM. https://www.cebm.net/2016/05/ocebm-levels-of-evidence/. Accessed 10 Nov 2019
