# Supplementary material for: Intravenous contrast medium extravasation: systematic review and updated ESUR Contrast Media Safety Committee Guidelines
Source: Eur Radiol. 2022 Feb 17;32(5):3056–66. doi: 10.1007/s00330-021-08433-4 (PMC9038843; doi:10.1007/s00330-021-08433-4)
Supplement: Supplementary file 4 — Supplementary file4 (DOCX 90.8 KB) [file 330_2021_8433_MOESM4_ESM.docx]

**Appendix 4: Characteristics, risk of bias assessment and level of evidence of included studies.**

| **Study, year** | **Study design** | **Population** | **Number of CMEX events/Total number of injections (%)** | **Variables studied and outcomes reported** | **Overall quality rating*** | **Level of evidence**** |
| --- | --- | --- | --- | --- | --- | --- |
| Miles et al, 1990[1] | Observational | Adult and children >12 years | 6 / 5280 (0.11) | CMEX events | Good | 4 |
| Hastings-Tolsma et al, 1993[2] | Randomized controlled trial | Adults | 18 / 18 (100.00) | Pain intensity, surface area of induration and erythema, and interstitial fluid volume | Poor | 2b |
| Cohan et al, 1996[3] | Review article | Not applicable | Not applicable | Not applicable | Fair | 5 |
| Federle et al, 1998[4] | Cohort | Adults | 48 / 5106 (0.90) | Injection rates, CMEX events, management | Fair | 4 |
| Jacobs et al, 1998[5] | Prospective | Adults | 41 / 6660 (0.62) | Extravasation rates, site of cannulation, type of CM used, catheter size and injection rates. | Fair | 4 |
| Nelson et al, 1998[6] | Observational study | Adults | Not applicable | Skin impedance | Poor | 5 |
| Birnbaum et al, 1999[7] | Prospective, non-blinded | Adults | 4 / 500 (0.8) | Types of CM used, Use of EDA in preventing CMEX | Fair | 4 |
| Powell et al, 2000[8] | Prospective | Adults | Not applicable | Sensitivity of using of EDA to detect CMEX | Poor | 4 |
| Vandeweyer et al, 2000[9] | Case-series | Adults | Not applicable | Outcomes of emergency suction in CMEX | Fair | 4 |
| Bellin et al, 2002[10] | Review article | Adults | Not applicable | Guidelines on treatment and management | Good | 2a |
| Khan and Holmes, 2002[11] | Quality improvement project | Adults | Not applicable | Use of flush-out technique in management of CMEX. | Fair | 4 |
| Sinan et al, 2005[12] | Prospective, non-randomised | Adults | 11 / 3560 (0.3) | Non-ionic CM, manual vs. power injector | Fair | 3b |
| Amaral et al, 2006[13] | Prospective | Children | 2 / 557 (0.3) | Age, weight, catheter size, location of venous access, flow rate, CM volume given, maximum injector pressure and incidence of CMEX. | Good | 4 |
| Namasivayam et al, 2006[14] | Review article | Adults and children | Not applicable | Suggested guidelines on diagnosis and management of CMEX | Poor | 5 |
| Selek et al, 2007[15] | Case report | Adult | Not applicable | Fasciotomy | Fair | 4 |
| Tsai et al, 2007[16] | Case-series | Adults | 8/8 (100) | Squeeze manoeuvre | Fair | 4 |
| Fallscheer et al, 2007[17] | Retrospective | Adults | 16/25000 (0.06) | Reasons, incidence, treatment and follow-up of CMEX. | Good | 2b |
| Wang et al, 2007[18] | Retrospective | Adults and children | 475 / 69657 (0.7) | Non-ionic CM, frequency, injection technique and management | Good | 4 |
| Schaverian et al, 2008[19] | Review article | Adults and children | Not applicable | Clinical presentation, risk factors and management of CMEX. | Good | 3a |
| Schwab et al, 2009[20] | Prospective | Adults  And in-vitro experiment | 2 / 58 (3.5) | Comparison of catheter gauges on CMEX and flow rates. | Fair | 4 |
| Sbitany et al, 2010[21] | Retrospective | Adults | Not applicable | Ionic vs. non-ionic CM on incidence and management outcomes of CMEX | Fair | 4 |
| Schummer et al, 2010[22] | Case-report | Adult | Not applicable | Multi-lumen CVC associated CMEX | Fair | 4 |
| Wienbeck et al, 2010[23] | Prospective | Adults | 52 / 4457 (1.2) | Non-ionic CM, frequency and type of injection site complications with use of automated power injector | Good | 4 |
| Belzunegui et al, 2011[24] | Case-report | Adult | Not applicable | Fasciotomy | Fair | 4 |
| Wilson, 2011[25] | Review article | Adult | Not applicable | Treatments of CMEX associated compartment syndrome | Fair | 5 |
| Alexander & Morrison, 2012 [26] | Retrospective | Adults | Port-a-cath: 0/307 (0) | Rates of CMEX related to Port-a-cath and comparing to PICC, CVCs and PVCs | Fair | 4 |
| Kingston et al, 2012[27] | Prospective, non-randomised | Adults | 119 / 26854 (0.44) | Non-ionic CM, Type of CT study, cannula size, rate of CM injection, personnel involved in cannulation | Fair | 4 |
| Lambeth et al, 2012[28] | Case-series | Adults | Not applicable | Complications of PICC lines | Fair | 4 |
| Davenport et al, 2012[29] | Retrospective | Adults and children | 78 / 24820 (0.31) | Non-ionic CM, effect of warming of CM on rates and volume of CMEX | Fair | 4 |
| Tonolini et al, 2012[30] | Review article | Adults and children | Not applicable | Incidence, mechanisms, diagnosis, risk factors, prevention, and treatment of CMEX | Fair | 4 |
| Moreno et al, 2013[31] | Retrospective | Adults | 330 / 118970 (0.28) | Non-ionic CM, effect of catheter gauge, dwell time, location, rate of administration, age and gender on rates and volume CMEX. | Fair | 4 |
| Johnson et al, 2014[32] | Randomised controlled trial | Adults | 20G non-fenestrated catheter: 2 / 102 (2.0)  18G fenestrated catheter: 0/103 (0) | Comparing performance of 20G non-fenestrated vs. 18G fenestrated catheters. | Good | 1b |
| Niv et al, 2014[33] | Retrospective and prospective | Adults and children | 83 / 37788 (0.48) | Non-ionic CM, catheter size, type of procedure, location, flow rate, gender, age and location of insertion on rates, symptoms, severity, treatments, volume and management outcomes of CMEX. | Good | 4 |
| Pacheco Compana et al, 2014[34] | Review article | Adults | Not applicable | Incidence, mechanisms, diagnosis, risk factors, prevention and management protocol of CMEX. | Poor | 4 |
| Shaqdan et al, 2014[35] | Retrospective | Adults and children | Non-ionic: 451 / 352125 (0.13)  GBCAs: 90 / 150266 (0.06) | Non-ionic and GBCAs. CT vs. MRI, power-injector vs manual, age, gender and setting, | Good | 4 |
| Alami et al, 2015[36] | Prospective | Adults | 18 / 2000 (0.9) | Incidence, age, site, rate, patient factors, type of CT scan relationship to CMEX. | Fair | 4 |
| Beckett et al, 2015[37] | Review article | Adults | Not applicable | Diagnosis, risk factors and management of CMEX. | Fair | 4 |
| Dykes et al, 2015[38] | National registry and quality improvement study | Adults | 1085 / 454497 (0.24) | Incidence, volume, severity of CMEX. Effect of a quality improvement initiative across multiple sites across the USA. | Good | 3a |
| Rose & Choi, 2015[39] | Review article | Adults | Not applicable | Discussion on diagnosis, risk factors and management of CMEX. | Poor | 4 |
| Nicola et al, 2016[40] | Guidelines | Adults | Not applicable | Guidelines on management of CMEX. | Poor | 4 |
| Sakellariou et al, 2016[41] | Computer simulation | Not applicable | Not applicable | Effect of CM viscosity and catheter tip position on vessel wall shear stress and risk of CMEX. | Good | 5 |
| Tonolini, 2016[42] | Letter | Adults and children | Not applicable | Discussion on value of radiographic assessment of CMEX. | Fair | 5 |
| Tamura et al, 2016[43] | Randomised controlled trial | Adults | 2 / 174 (1.1) | 24G side-holes catheter vs.  22G end-hole catheter impact on CMEX, tolerance for differing pressures, flow rates and image quality. | Fair | 1b |
| Rupp et al, 2016[44] | Retrospective | Adults | 115 / 40143 (0.3) | Comparison between rates of CMEX with ultrasound guided IV insertion vs standard peripheral IV insertion. | Fair | 3b |
| Kok et al, 2016[45] | Randomised controlled trial | Adults | 0 / 608 (0) | Comparison of different flow rates and CM concentrations on CMEX, patient comfort and image quality of CTCA. | Good | 1b |
| Cleary et al, 2017[46] | Survey | Not applicable | Not applicable | National survey of management protocols of CMEX. | Good | 5 |
| Connor et al, 2017[47] | Quality improvement study | Adults | Not applicable | Characteristics of CMEX injury and CMEX rates pre and post-intervention. | Good | 3a |
| Kim et al, 2017[48] | Retrospective | Adults | 80 / 218000 (0.04) | Incidence, anatomical location, age, flow rates, gauge of catheter and volume of CMEX. Also experience of using multiple slit technique as treatment of CMEX. | Good | 3b |
| Craigie et al, 2018[49] | Case-series | Adults | Not applicable | Tip migration with use of PICC line with automated pressure injectors. | Fair | 4 |
| Ding et al, 2018[50] | Systematic and meta-analysis | Adults and children | Not applicable | Impact on volume and frequency of CMEX was assessed by gender, cannula type/size, infusion rate, US guided insertion of PVC, type of venous access, warming of CM, type of healthcare professional inserting PVC, dwell time of PVC, quality improvement project and use of EDA. | Good | 1a |
| Behzadi et al, 2018[51] | Systematic review | Adults and children | 2191 / 1104872 (0.2%) | Impact on volume, severity and frequency of CMEX assessed by GBCAs vs. iodinated CM, gender, age, type of healthcare professional inserting PVC, catheter location, catheter size, power vs manual injection, injection rate, in-patients vs. outpatients and CM temperature. | Good | 2a |
| Hwang et al, 2018[52] | Retrospective | Adults | 321 / 142651 (0.23) | Frequency and outcomes of CMEX relationship with gender, age, hospitalisation status, CM viscosity and injection rate. | Good | 3b |
| Sonis et al, 2018[53] | Retrospective | Adults | Unknown | Comparison of different management strategies of CMEX. | Good | 4 |
| Hrycyk et al, 2018[54] | Review article | Adults | Not applicable | Frequency, risk factors, symptoms and treatments of CMEX. | Poor | 4 |
| Ko et al, 2018[55] | Prospective | Adults | 27 / 67129 (0.04) | Role of age, injection method, different preventative techniques and management of CMEX. | Good | 2b |
| Mandlik et al, 2019[56] | Review article | Adults | Not applicable | Incidence, risk factors, management and suggested protocol of CMEX. | Poor | 3a |
| Barrera et al, 2019[57] | Retrospective | Children | 18 / 2429 (0.7) | Age, height, weight, gender, manual vs. power-injector, type of CM, injection site, flow rate, volume of CM used, peak pressure, catheter size impact on frequency, severity and need for surgical consult in relation to CMEX. | Good | 3b |
| Raveendran et al, 2019[58] | Case-series | Adults | Not applicable | Use of multiple stab incisions and evacuation technique in CMEX. | Fair | 4 |
| Favot et al, 2019[59] | Retrospective | Adults | 74 / 29508 (0.2) | CMEX rates for nurse-led placement of ultrasound guided IV catheters. Demographic factors are also studied association with CMEX. | Good | 3b |
| Stroeder et al, 2020 [60] | Prospective | Adults | 25 / 3514 (0.71) | Complication rates, including CMEX, with use of Iomeprol 400 injections for CT through various sized cannulas. Additional significant finding suggesting aspiration test should be performed before each injection. | Good | 2b |
| Van Veelen et al, 2020 [61] | Case report and literature review | Adults | Not applicable | Descriptive analysis of 12 case reports of compartment syndrome due to CMEX. | Good | 4 |
| **Study Quality Assessment Tools. National Heart, Lung and Blood Institute, National Institutes of Health.[62]* | | | | | | |
| *** Levels of Evidence. OCEBM Levels of Evidence Working Group*. “The Oxford Levels of Evidence 2”. [63]* | | | | | | |

1. Miles SG, Rasmussen JF, Litwiller T, Osik A (1990) Safe use of an intravenous power injector for CT: experience and protocol. Radiology 176:69–70. https://doi.org/10.1148/radiology.176.1.2353114

2. Hastings-Tolsma MT, Yucha CB, Tompkins J, et al (1993) Effect of warm and cold applications on the resolution of i.v. infiltrations. Res Nurs Health 16:171–178

3. Cohan RH, Bullard MA, Ellis JH, et al (1997) Local reactions after injection of iodinated contrast material: detection, management, and outcome. Acad Radiol 4:711–718

4. Federle MP, Chang PJ, Confer S, Ozgun B (1998) Frequency and effects of extravasation of ionic and nonionic CT contrast media during rapid bolus injection. Radiology 206:637–640. https://doi.org/10.1148/radiology.206.3.9494479

5. Jacobs JE, Birnbaum BA, Langlotz CP (1998) Contrast media reactions and extravasation: relationship to intravenous injection rates. Radiology 209:411–416. https://doi.org/10.1148/radiology.209.2.9807567

6. Nelson RC, Anderson FA, Birnbaum BA, et al (1998) Contrast media extravasation during dynamic CT: detection with an extravasation detection accessory. Radiology 209:837–843. https://doi.org/10.1148/radiology.209.3.9844684

7. Birnbaum BA, Nelson RC, Chezmar JL, Glick SN (1999) Extravasation detection accessory: clinical evaluation in 500 patients. Radiology 212:431–438. https://doi.org/10.1148/radiology.212.2.r99au14431

8. Powell CC, Li J ming, Rodino L, Anderson FA (2000) A New Device to Limit Extravasation During Contrast-Enhanced CT. Am J Roentgenol 174:315–318. https://doi.org/10.2214/ajr.174.2.1740315

9. Vandeweyer E, Heymans O, Deraemaecker R (2000) Extravasation injuries and emergency suction as treatment. Plast Reconstr Surg 105:109–110. https://doi.org/10.1097/00006534-200001000-00019

10. Bellin M-F, Jakobsen JA, Tomassin I, et al (2002) Contrast medium extravasation injury: guidelines for prevention and management. Eur Radiol 12:2807–2812. https://doi.org/10.1007/s00330-002-1630-9

11. Khan MS, Holmes JD (2002) Reducing the morbidity from extravasation injuries. Ann Plast Surg 48:628–632; discussion 632. https://doi.org/10.1097/00000637-200206000-00011

12. Sinan T, Al-Khawari H, Chishti FA, et al (2005) Contrast media extravasation: manual versus power injector. Med Princ Pract Int J Kuwait Univ Health Sci Cent 14:107–110. https://doi.org/10.1159/000083921

13. Amaral JG, Traubici J, BenDavid G, et al (2006) Safety of power injector use in children as measured by incidence of extravasation. AJR Am J Roentgenol 187:580–583. https://doi.org/10.2214/AJR.05.0667

14. Namasivayam S, Kalra MK, Torres WE, Small WC (2006) Adverse reactions to intravenous iodinated contrast media: an update. Curr Probl Diagn Radiol 35:164–169. https://doi.org/10.1067/j.cpradiol.2006.04.001

15. Selek H, Ozer H, Aygencel G, Turanli S (2007) Compartment syndrome in the hand due to extravasation of contrast material. Arch Orthop Trauma Surg 127:425–427. https://doi.org/10.1007/s00402-006-0238-y

16. Tsai YS, Cheng SM, Ng SP, et al (2007) Squeeze maneuver: an easy way to manage radiological contrast-medium extravasation. Acta Radiol Stockh Swed 1987 48:605–607. https://doi.org/10.1080/02841850701324094

17. Fallscheer P, Kammer E, Roeren T, Meuli-Simmen C (2007) Injury to the upper extremity caused by extravasation of contrast medium: a true emergency. Scand J Plast Reconstr Surg Hand Surg 41:26–32

18. Wang CL, Cohan RH, Ellis JH, et al (2007) Frequency, management, and outcome of extravasation of nonionic iodinated contrast medium in 69,657 intravenous injections. Radiology 243:80–87. https://doi.org/10.1148/radiol.2431060554

19. Schaverien MV, Evison D, McCulley SJ (2008) Management of large volume CT contrast medium extravasation injury: technical refinement and literature review. J Plast Reconstr Aesthetic Surg JPRAS 61:562–565; discussion 565. https://doi.org/10.1016/j.bjps.2007.02.024

20. Schwab SA, Uder M, Anders K, et al (2009) Peripheral intravenous power injection of iodinated contrast media through 22G and 20G cannulas: can high flow rates be achieved safely? A clinical feasibility study. ROFO Fortschr Geb Rontgenstr Nuklearmed 181:355–361. https://doi.org/10.1055/s-0028-1109181

21. Sbitany H, Koltz PF, Mays C, et al (2010) CT contrast extravasation in the upper extremity: strategies for management. Int J Surg Lond Engl 8:384–386. https://doi.org/10.1016/j.ijsu.2010.06.002

22. Schummer C, Sakr Y, Steenbeck J, et al (2010) Risk of extravasation after power injection of contrast media via the proximal port of multilumen central venous catheters: case report and review of the literature. ROFO Fortschr Geb Rontgenstr Nuklearmed 182:14–19. https://doi.org/10.1055/s-0028-1109742

23. Wienbeck S, Fischbach R, Kloska SP, et al (2010) Prospective study of access site complications of automated contrast injection with peripheral venous access in MDCT. AJR Am J Roentgenol 195:825–829. https://doi.org/10.2214/AJR.09.3739

24. Belzunegui T, Louis CJ, Torrededia L, Oteiza J (2011) Extravasation of radiographic contrast material and compartment syndrome in the hand: a case report. Scand J Trauma Resusc Emerg Med 19:9. https://doi.org/10.1186/1757-7241-19-9

25. Wilson BG (2011) Contrast media-induced compartment syndrome. Radiol Technol 83:63–77

26. Alexander MD, Morrison HL (2012) Power-injectable ports: safety during placement, therapeutic use, and contrast administration during computed tomography procedures. J Vasc Access 13:432–437. https://doi.org/10.5301/jva.5000074

27. Kingston RJ, Young N, Sindhusake DP, Truong M (2012) Study of patients with intravenous contrast extravasation on CT studies, with radiology staff and ward staff cannulations. J Med Imaging Radiat Oncol 56:163–167. https://doi.org/10.1111/j.1754-9485.2012.02355.x

28. Lambeth L, Goyal A, Tadros A, et al (2012) Peripherally inserted central catheter tip malposition caused by power contrast medium injection. J Vasc Interv Radiol JVIR 23:981–983. https://doi.org/10.1016/j.jvir.2012.04.024

29. Davenport MS, Wang CL, Bashir MR, et al (2012) Rate of Contrast Material Extravasations and Allergic-like Reactions: Effect of Extrinsic Warming of Low-Osmolality Iodinated CT Contrast Material to 37°C. Radiology 262:475–484. https://doi.org/10.1148/radiol.11111282

30. Tonolini M, Campari A, Bianco R (2012) Extravasation of radiographic contrast media: prevention, diagnosis, and treatment. Curr Probl Diagn Radiol 41:52–55. https://doi.org/10.1067/j.cpradiol.2011.07.004

31. Moreno CC, Pinho D, Nelson RC, et al (2013) Lessons learned from 118,970 multidetector computed tomographic intravenous contrast material administrations: impact of catheter dwell time and gauge, catheter location, rate of contrast material administration, and patient age and sex on volume of extravasate. J Comput Assist Tomogr 37:286–288. https://doi.org/10.1097/RCT.0b013e31828211da

32. Johnson PT, Christensen GM, Fishman EK (2014) I.v. contrast administration with dual source 128-MDCT: a randomized controlled study comparing 18-gauge nonfenestrated and 20-gauge fenestrated catheters for catheter placement success, infusion rate, image quality, and complications. AJR Am J Roentgenol 202:1166–1170. https://doi.org/10.2214/AJR.13.11730

33. Niv G, Costa M, Kicak P, Richman K (2014) Vascular extravasation of contrast medium in radiological examinations: University of California San Diego Health System Experience. J Patient Saf 10:105–110. https://doi.org/10.1097/PTS.0000000000000114

34. Pacheco Compaña FJ, Gago Vidal B, Méndez Díaz C (2014) [Extravasation of contrast media at the puncture site: Strategies for managment]. Radiologia 56:295–302. https://doi.org/10.1016/j.rx.2014.02.003

35. Shaqdan K, Aran S, Thrall J, Abujudeh H (2014) Incidence of contrast medium extravasation for CT and MRI in a large academic medical centre: a report on 502,391 injections. Clin Radiol 69:1264–1272. https://doi.org/10.1016/j.crad.2014.08.004

36. Alami Z, Nasri S, Ahid S, Kacem HH (2015) Extravasation of contrast medium during CT examination: an observational case-control study. Pan Afr Med J 20:. https://doi.org/10.11604/pamj.2015.20.89.3276

37. Beckett KR, Moriarity AK, Langer JM (2015) Safe Use of Contrast Media: What the Radiologist Needs to Know. Radiogr Rev Publ Radiol Soc N Am Inc 35:1738–1750. https://doi.org/10.1148/rg.2015150033

38. Dykes TM, Bhargavan-Chatfield M, Dyer RB (2015) Intravenous contrast extravasation during CT: a national data registry and practice quality improvement initiative. J Am Coll Radiol JACR 12:183–191. https://doi.org/10.1016/j.jacr.2014.07.021

39. Rose TA, Choi JW (2015) Intravenous Imaging Contrast Media Complications: The Basics That Every Clinician Needs to Know. Am J Med 128:943–949. https://doi.org/10.1016/j.amjmed.2015.02.018

40. Nicola R, Shaqdan KW, Aran S, et al (2016) Contrast Media Extravasation of Computed Tomography and Magnetic Resonance Imaging: Management Guidelines for the Radiologist. Curr Probl Diagn Radiol 45:161–164. https://doi.org/10.1067/j.cpradiol.2015.08.004

41. Sakellariou S, Li W, Paul MC, Roditi G (2016) Rôle of contrast media viscosity in altering vessel wall shear stress and relation to the risk of contrast extravasations. Med Eng Phys 38:1426–1433. https://doi.org/10.1016/j.medengphy.2016.09.016

42. Tonolini M (2016) Contrast Medium Extravasation: The Importance of Radiographic Assessment. Curr Probl Diagn Radiol 45:236–237. https://doi.org/10.1067/j.cpradiol.2016.02.002

43. Tamura A, Kato K, Kamata M, et al (2017) Selection of peripheral intravenous catheters with 24-gauge side-holes versus those with 22-gauge end-hole for MDCT: A prospective randomized study. Eur J Radiol 87:8–12. https://doi.org/10.1016/j.ejrad.2016.12.005

44. Rupp JD, Ferre RM, Boyd JS, et al (2016) Extravasation Risk Using Ultrasound-guided Peripheral Intravenous Catheters for Computed Tomography Contrast Administration. Acad Emerg Med Off J Soc Acad Emerg Med 23:918–921. https://doi.org/10.1111/acem.13000

45. Kok M, Mihl C, Hendriks BMF, et al (2016) Patient Comfort During Contrast Media Injection in Coronary Computed Tomographic Angiography Using Varying Contrast Media Concentrations and Flow Rates: Results From the EICAR Trial. Invest Radiol 51:810–815. https://doi.org/10.1097/RLI.0000000000000284

46. Cleary N, McNulty JP, Foley SJ, Kelly E (2017) An investigation into current protocols and radiographer opinions on contrast extravasation in Irish CT departments. Radiogr Lond Engl 1995 23:e87–e92. https://doi.org/10.1016/j.radi.2017.05.009

47. Conner B, Ash R, Allen W, et al (2017) Preventing Intravenous Contrast Extravasation in CT: A Simple Solution. J Am Coll Radiol JACR 14:1326–1332. https://doi.org/10.1016/j.jacr.2017.05.020

48. Kim SM, Cook KH, Lee IJ, et al (2017) Computed tomography contrast media extravasation: treatment algorithm and immediate treatment by squeezing with multiple slit incisions. Int Wound J 14:430–434. https://doi.org/10.1111/iwj.12628

49. Craigie M, Meehan L, Harper J (2018) Tip Migration Post-Contrast Pressure Injection Through Pressure-Injectable Peripherally Inserted Central Catheters Causing Vascular Injury: A Report of 3 Cases. Cardiovasc Intervent Radiol 41:509–512. https://doi.org/10.1007/s00270-017-1828-5

50. Ding S, Meystre NR, Campeanu C, Gullo G (2018) Contrast media extravasations in patients undergoing computerized tomography scanning: a systematic review and meta-analysis of risk factors and interventions. JBI Database Syst Rev Implement Rep 16:87–116. https://doi.org/10.11124/JBISRIR-2017-003348

51. Heshmatzadeh Behzadi A, Farooq Z, Newhouse JH, Prince MR (2018) MRI and CT contrast media extravasation. Medicine (Baltimore) 97:. https://doi.org/10.1097/MD.0000000000010055

52. Hwang EJ, Shin C-I, Choi YH, Park CM (2018) Frequency, outcome, and risk factors of contrast media extravasation in 142,651 intravenous contrast-enhanced CT scans. Eur Radiol 28:5368–5375. https://doi.org/10.1007/s00330-018-5507-y

53. Sonis JD, Gottumukkala RV, Glover M, et al (2018) Implications of iodinated contrast media extravasation in the emergency department. Am J Emerg Med 36:294–296. https://doi.org/10.1016/j.ajem.2017.11.012

54. Hrycyk J, Heverhagen JT, Boehm I (2019) What you should know about prophylaxis and treatment of radiographic and magnetic resonance contrast medium extravasation. Acta Radiol Stockh Swed 1987 60:496–500. https://doi.org/10.1177/0284185118782000

55. Ko C-H, Tay SY, Chang H-C, Chan WP (2018) Large-volume iodinated contrast medium extravasation: low frequency and good outcome after conservative management in a single-centre cohort of more than 67,000 patients. Eur Radiol 28:5376–5383. https://doi.org/10.1007/s00330-018-5514-z

56. Mandlik V, Prantl L, Schreyer AG (2019) Contrast Media Extravasation in CT and MRI - A Literature Review and Strategies for Therapy. ROFO Fortschr Geb Rontgenstr Nuklearmed 191:25–32. https://doi.org/10.1055/a-0628-7095

57. Barrera CA, White AM, Shepherd AM, et al (2019) Contrast Extravasation using Power Injectors for Contrast-Enhanced Computed Tomography in Children: Frequency and Injury Severity. Acad Radiol. https://doi.org/10.1016/j.acra.2019.04.008

58. Raveendran S, Rajendra Benny K, Monica S, et al (2019) Multiple Stab Incisions and Evacuation Technique for Contrast Extravasation of the Hand and Forearm. J Hand Surg 44:71.e1-71.e5. https://doi.org/10.1016/j.jhsa.2018.08.009

59. Favot M, Gallien J, Malik A, et al (2019) Contrast Extravasation as a Complication of Emergency Nurse-Performed Ultrasound-Guided Peripheral Intravenous Catheter Placement. J Emerg Nurs 45:512–516. https://doi.org/10.1016/j.jen.2019.05.016

60. Stroeder J, Fries P, Raczeck P, et al (2020) Prospective safety evaluation of automated iomeprol 400 injections for CT through peripheral venous cannulas. Clin Radiol 75:396.e1-396.e6. https://doi.org/10.1016/j.crad.2019.12.013

61. van Veelen NM, Link B-C, Donner G, et al (2020) Compartment syndrome of the forearm caused by contrast medium extravasation: A case report and review of the literature. Clin Imaging 61:58–61. https://doi.org/10.1016/j.clinimag.2020.01.013

62. National Heart, Lung, and Blood Institute. (2019). Study Quality Assessment Tools. https://www.nhlbi.nih.gov/health-topics/study-quality-assessment-tools. Accessed 6 Sep 2020

63. OCEBM Levels of Evidence. (2016) In: CEBM. https://www.cebm.net/2016/05/ocebm-levels-of-evidence/. Accessed 10 Nov 2019
