# Supplementary material for: Intravenous contrast medium extravasation: systematic review and updated ESUR Contrast Media Safety Committee Guidelines
Source: Eur Radiol. 2022 Feb 17;32(5):3056–66. doi: 10.1007/s00330-021-08433-4 (PMC9038843; doi:10.1007/s00330-021-08433-4)
Supplement: Supplementary file 5 — Supplementary file5 (DOCX 33.3 KB) [file 330_2021_8433_MOESM5_ESM.docx]

**Appendix 5: A summary of the different EDA devices which have been tested clinically**

| **Devices** | **Description** |
| --- | --- |
| MEDRAD Stellant XDS (Bayer)  *Warrendale, PA, USA* | This system uses a radiofrequency device for detection and warns the operator to the presence of extravasation[1]. A single case-series of 25 adults who were considered high risk for CMEX underwent CT angiography and relevant data was collected. Overall, the device alerted to five episodes of extravasation with no more than 8 ml of CM introduced[2]. One disadvantage to the use of this device is that it takes up to 3 minutes to set-up which may be considered an obstacle for day-to-day use. |
| SimpliDetect (Neorad AS)  *Roosendaal, Netherlands* | This device uses continuous wave Doppler ultrasound via a sensor placed distal to the cannula insertion site to monitor blood flow and detect a correct injection by a strong increase in Doppler signal strength and velocity. The device is linked to the injector and interrupts injection in the event of complication, amongst other errors (e.g. equipment failure and inadvertent air injections). The device detected one extravasation and four other unrelated problems in a study of 198 patients in a multi-centre study[3]. |
| E-Z EM^TM^ Empower CT^(R)^ (Bracco)  *Westbury, NY, USA* | This system uses electrode patches attached to the skin above the injection site[4] and detects changes in skin electrical impedance for the identification of extravasation. In a prospective, multi-institutional study of 500 patients, this device detected >10 ml extravasation with 100% sensitivity and 98% specificity[5]. A further study which delivered 0.9% sodium chloride or 5% dextrose instead of CM via a power injector at differing rates showed similar results with alerting to extravasation of no more than 13 ml with high sensitivity and specificity[6]. A purported advantage of this device is that it is relatively quick to set-up, only taking approximately 20 seconds. |
| LD Contrast Agent Leak Detection Support System (Nemoto Kyorindo)  *Tokyo, Japan* | This device utilises infrared rays to detect extravasation. A quality improvement project by Teo et al. has shown that together with other interventions CMEX reduced with the use of this device[7]. However, more prospective research is needed to assess the weight of effect the detection system has on reducing CMEX and its severity. |

1. Radiology - Medrad Stellant. http://radiology.bayer.co.uk/products/ct/medrad-stellant/. Accessed 13 Oct 2019

2. Saade C, Brennan P (2011) Clinical Implementation of the New MEDRAD XDS Contrast Extravasation Detector for Multidetector Computed Tomography. J Med Imaging Radiat Sci 42:179–182. https://doi.org/10.1016/j.jmir.2011.05.003

3. Hoff L, Brabrand K, Andersen NB, Medhus S (2008) Monitoring X-ray contrast agent injections with Doppler ultrasound. 2008 IEEE Ultrason Symp 13–16. https://doi.org/10.1109/ultsym.2008.0004

4. Bracco EZEM Empower CT & Empower CTA-D 200ml/200ml CT Contrast Media Injectors Syringes. In: Dispos. Syr. Suppliers Medrad CT MRI ANGIO Contrast Inject. https://www.medismed.com/product/bracco-ezem-empower-ct-empower-cta-dual-head-injectors/. Accessed 13 Oct 2019

5. Birnbaum BA, Nelson RC, Chezmar JL, Glick SN (1999) Extravasation detection accessory: clinical evaluation in 500 patients. Radiology 212:431–438. https://doi.org/10.1148/radiology.212.2.r99au14431

6. Powell CC, Li J ming, Rodino L, Anderson FA (2000) A New Device to Limit Extravasation During Contrast-Enhanced CT. Am J Roentgenol 174:315–318. https://doi.org/10.2214/ajr.174.2.1740315

7. Teo M, Ong C, Ying A, Hng M (2015) Extravasation of Contrast Medium during CT Scanning – Tracking and Reduction of Rate of Extravasation. https://posterng.netkey.at/esr/viewing/index.php?module=viewing_poster&task=viewsection&pi=126853&ti=422983&si=1478&searchkey=. Accessed 16 Oct 2019
